# Loneliness in Old Age, the Related Factors, and Its Association with Demographics and Districts of Residence

**DOI:** 10.3390/ijerph18179398

**Published:** 2021-09-06

**Authors:** Susan Ka Yee Chow, Florence M. F. Wong, Edward Kwok Yiu Choi

**Affiliations:** 1 School of Nursing, Tung Wah College, Hong Kong; florencewong@twc.edu.hk; 2 Department of Anaesthesiology, The University of Hong Kong, Hong Kong; edwardkyc@gmail.com

**Keywords:** loneliness, self-rated health, activities of daily living, living districts

## Abstract

Loneliness among older people has now become a serious public health issue. There have been few previous studies conducted among Chinese populations on the correlations between loneliness, self-rated health, and instrumental activities of daily living (IADL), and their association with demographic characteristics. In this study, data were collected using quota sampling through survey interviews. Older people living in representative districts were recruited. Of the participants, 60.1% rated their health as average and 58.1% showed a high level of loneliness. IADL and self-rated health (SRH) were found to be moderately positively correlated, with r = 0.357, *p* < 0.001. A low negative correlation was found between the level of loneliness and IADL, with r = −0.276; and SRH, with r = −0.288, *p* < 0.05. Ordinal Regression results showed that subjects with higher IADL scores (OR: 0.64, 95% CI: 0.39–1.05) were less lonely, while those with a less desirable economic status (OR: 3.34, 95% CI: 1.40–7.96) and living in the central business district were more likely to have a higher loneliness score (OR: 21.33, 95% CI: 4.81–95.41). It is essential to screen for loneliness, and interventions should be focused on improving social connections and support for older people to overcome their feelings of loneliness.

## 1. Introduction

Loneliness among older people has become a serious public health concern. Loneliness is generally defined as a subjective feeling of distress from being isolated or alone due to the individual’s perception of a lack of social interaction or poor social relationships [1,2]. According to the U.S. Centers for Disease Control and Prevention, older adults suffering from loneliness and social isolation are at an increased risk of dementia, depression, and anxiety [3]. Older people with a variety of physical health problems, such as cognitive decline, cardiovascular diseases, Alzheimer’s disease, and inflammatory diseases may be at a higher risk of experiencing loneliness [4,5,6,7], and of developing mental health problems such as anxiety and depression [5]. Loneliness might also have negative effects on the structure of the brain, by affecting the volume of grey matter in the brain [8]. The level of loneliness can be significantly higher for older people who have trouble attending to their activities of daily living because they are completely or partially dependent on others [9].

The trend of ageing is becoming a major concern for public health authorities worldwide. According to the Census and Statistics Department, Hong Kong, at present, one out of eight people in Hong Kong is aged 65 or above. The older population is projected to further increase to 31% in 2036 and 37% in 2066 [10]. Another concern is that the number of solitary older people has been increasing continuously. In 2016, there were approximately 150,000 solitary older people (aged 65 or above) in Hong Kong and the number has increased from 11.6% to 13.1% in the past decade [11]. Older people who are living alone tend to experience higher levels of loneliness [12]. This could be related to inadequate family support, social support, or interactions among older adults [13].

Most current studies on loneliness have been conducted in Western countries, while studies about loneliness among older people in Chinese populations have been comparatively sparse, especially in Hong Kong. Since the culture, social environment, and family values in Western and Chinese societies differ, perceptions of loneliness in those societies may also differ. There is evidence to show that an individual’s cultural background affects that person’s experience of loneliness [14]. People in individualistic societies have been reported to be lonelier than those in collectivist societies [15]. However, the opposite has also been reported, with levels of loneliness said to be higher in collectivist than in individualistic societies [16]. Owing to the collective culture of the Chinese, older people in Hong Kong may experience more loneliness due to reduced interactions with family members, as most young couples opt to form a nuclear family.

Current studies suggest that socio-demographic characteristics, and physical and psychosocial health status are contributing to loneliness among older people. The common socio-demographic factors associated with loneliness are age, gender, living conditions, marital status, health status, social relationship, and financial status [17,18]. Furthermore, loneliness has been correlated with poor Self-rated Health (SRH). A study indicated that older adults in Hong Kong with poor SRH showed higher levels of loneliness [19]. By contrast, older adults who perceived themselves as being in good health seldom reported experiencing feelings of loneliness [20]. Loneliness is also correlated with living status, with older people who were living alone and with poor SRH, being at an increased risk of feeling lonely [21]. As loneliness is strongly correlated with chronic illnesses and a deficiency in leisure activities, it may have a negative effect on the quality of life of older adults [17]. Older adults with chronic diseases tend to be on long-term medications. A path analysis showed that social isolation and loneliness had a negative indirect effect on medication adherence, while social support and loneliness fully mediated the relationship between social isolation and medication [22].

The Instrumental Activities of Daily Living (IADLs) are complex activities related to one’s ability to live independently in the community. A person’s ability to use a phone, go shopping, prepare food, do laundry, maintain the cleanliness of their home, take care of their finances, use transportation, and adhere to medications is closely related to their ability to function socially and is essential to their ability to remain independent [23]. Chronic diseases, which could compromise an individual’s physical condition, could increase that person’s dependence and decrease his/her capabilities [24]. Other factors such as sex, age, literacy levels, and sociocultural involvement could influence a person’s capabilities in daily living [25].

Currently, there is a lack of studies on the association between loneliness and SRH among older people, as the focus has been on depression and quality of life. Most importantly, there has been no study on the correlation between loneliness, SRH, and IADL in association with demographic characteristics among older people in Hong Kong. In view of the negative impacts of loneliness on both physical and mental well-being, this study increases knowledge on the relationships between self-perceived health, socio-demographic factors, and loneliness. The new knowledge could facilitate the development of interventions to reduce the loneliness of older people and ultimately promote their well-being and enhance their quality of life.

The aim of this study was to examine the relationship between loneliness, IADL, and SRH among community-dwelling older adults in Hong Kong. The objectives were: (1) to examine older people’s perceptions of their own health; (2) to examine the level of loneliness of older adults; (3) to examine the level of IADL of older adults; (4) to examine the correlation between SRH, loneliness, and IADL among older people; and (5) to identify the predictors of loneliness. Therefore, our research question was “Are there any relationships between loneliness, the instrumental activities of daily living, and self-rated health among community-dwelling older people in Hong Kong?”

## 2. Materials and Methods

A cross-sectional design was adopted in this study. Quota sampling, a nonprobability sampling method, was used because we focused on older people living in representative districts [26]. After the quotas were determined, convenience sampling was used for subject recruitment in each district. There were 150 participants who were invited, with 143 agreeing to respond. Each participant in the selected districts was asked to answer a questionnaire delivered face-to-face at a single data collection point.

The six districts out of the eighteen districts in Hong Kong that were selected for sampling were chosen because a relatively high percentage of older people live in those districts. These six districts were: Wong Tai Sin, Kwun Tong, Island East, Kwai Tsing, Central and Island West, and Sha Tin [11]. The criteria for inclusion in this study were: (1) adults aged 65 years or older; (2) living in the community; and (3) able to communicate in Chinese or Cantonese. The exclusion criteria included: (1) those without adequate cognitive capability to communicate; and (2) those who were institutionalized.

### 2.1. Sample Size Calculation

With regard to the sample size, we assumed that the effect of the current study would be similar to that of a previous study by Yang et al. conducted in 2018 [9]. Through Cohen’s equation [27], the size of the sample required to achieve a significance level of 0.05 and a power of 80% was determined to be 114. Another estimation that included 20 predictors was assumed in the regression model, with R2 = 0.178 [9]. Taking into account a 20% dropout rate, 143 subjects were required for the present study.

### 2.2. Research Questionnaire

The section on demographic characteristics was developed by the researchers. The demographic factors included gender, age group, marital status, education level, economic status, accommodation status, living arrangement, and whether the individual had any chronic diseases. With regard to the economic status, four subjective descriptors instead of actual monetary value were used to illustrate economic status. This is because the interpretation of monetary value could differ from person to person and it is difficult for overseas readers to make comparisons.

#### 2.2.1. Self-Rated Health

A single question, “How would you rate your health in general?”, was used to measure SRH. A 5-point Likert scale was used, with 1 = very poor, 2 = poor, 3 = average, 4 = good, and 5 = excellent. The meta-analysis showed that this single-item health question was able to predict mortality risk. People who rated their health as “Poor” had double the risk of counterparts who rated their health as “Excellent” [28]. A recent study in 2018 confirmed that loneliness was having a significant negative relationship to self-rated health in middle-aged and older adults [29]. For the test-retest reliability, the Intra-class Correlation Coefficient of the present study was 0.86.

#### 2.2.2. Loneliness

The six-item De Jong Gierveld Loneliness Scale was adopted to measure levels of loneliness. The scale comprises 3 items to assess the level of emotional loneliness and 3 items for social loneliness [30]. A translated Hong Kong Chinese version of the scale with good validity and reliability was used in this study [31]. Sample items included “I experience a general sense of emptiness” and “There are enough people I can trust completely”. A 3-point Likert scale was used to measure each item. A few items were phrased negatively and scores were reversed. The overall loneliness score ranges from 0 to 6, where 0 means no loneliness and 6 indicates severe loneliness. The Cronbach’s α was 0.76 and the test-retest reliability with the Intra-class Correlation Coefficient was 0.98 in the present study.

#### 2.2.3. Instrumental Activities of Daily Living

The Instrumental Activities of Daily Living (IADL) scale developed by Lawton was used to measure the independent living skills of the older adults [23]. A nine-item Hong Kong Chinese version of the scale was adopted in this study [32]. A 4-point Likert scale (3 = without any help, 2 = can do it on my own but sometimes need help, 1 = need some help, and 0 = cannot do it on my own) was used to rate the subject’s ability to perform specific tasks. Total scores ranged from 0 to 27, with higher scores representing a higher capability to carry out independent living skills. The intra-class correlation of the scale in the present study was 0.967.

### 2.3. Statistical Analysis

Statistical analysis was performed using IBM SPSS Statistics for Windows, Version 26.0 (IBM Corp., Armonk, NY, USA computer software). Descriptive statistics were used for the demographic characteristics, the SRH, IADH, and De Jong Gierveld Loneliness Scale. A normality test was conducted to determine whether a parametric or nonparametric test was to be used. A correlation coefficient was used to examine the relationships between loneliness, SRH, and IADL. A one-way ANOVA or Kruskal–Wallis H test was conducted to examine the differences between three or more groups. An Ordinal Regression Analysis was conducted to identify the predictors of loneliness. To further elaborate on the results, the Odds Ratio and a 95% confidence interval were identified to explain the parameter estimates. The outputs of the Ordinal Regression were extracted to Microsoft Excel through a simple formula to calculate the Odds Ratio and the 95% Confidence Interval [33]. A *p*-value of <0.05 in a two-tailed test was considered statistically significant.

## 3. Results

### 3.1. Demographic Characteristics

There were more female (55.4%) than male subjects (45.5%). The majority of the subjects were within the age groups of 65–70 (34.3%) and 71–75 (32.9%). Most of them were married (51.7%) and had attained a primary level of education or below (46.2%). Most reported their economic status as just sufficient to meet their daily expenses (55.2%). As for their districts of residence, the distributions among the six districts were fairly even, ranging from 14.0 to 22.4%. Please refer to Table 1 for details.

### 3.2. The Level of SRH, Loneliness, and IADL of Older People

Of the participants, 60.1% rated their health as average and only 3.5% as very good, while 4.9% rated their health as very poor.

With regard to loneliness, the median score was five with an inter-quartile range of three. A total of 58.1% of the participants showed a high level of loneliness, with 32.2% scoring five and 25.9% scoring six. Only 8.4% of the participants did not feel lonely, with a score of 0–1.

In terms of the instrumental activities of daily living of older people, 14.7% the participants had a high level of independent living skills, with a maximum score of 27, while 58.7% had a score of between 19 and 26, indicating that they could accomplish most of the activities by themselves or sometimes needed help. Among the respondents, the highest score was 27 and the lowest score was one. The mean IADL score was 20.77 (SD 5.77). Please refer to Table 2, Table 3 and Table 4 for details.

### 3.3. Correlations between Loneliness, SRH, and IADL

The Spearman correlation coefficient (*r*) was used to examine the associations between IADL, SRH, and the level of loneliness. IADL and SRH were found to be moderately positively correlated, with *r* = 0.357 and *p* < 0.001. A low negative correlation was found between the level of loneliness and IADL, with *r* = −0.276; and SRH, with *r* = −0.292 and *p* < 0.05. Please refer to Table 5 for details.

### 3.4. Differences in Loneliness Level among Subjects Living in Different Districts

The Kruskal–Wallis H test showed a significant difference in the level of loneliness among the subjects living in the six districts, with **χ^2^** 18.87 and *p* = 0.002. A post-hoc test with Bonferroni corrections was conducted to examine the significant differences between the pairwise comparisons. The results indicated that there were significant differences between the subjects living in Shatin and their counterparts in Central and Island West (*p* < 0.001), and Kwun Tong (*p* = 0.003). Please refer to Table 6 and Table 7 for details.

An Ordinal Regression analysis was used to examine the predictors of loneliness. The independent variables were gender, age group, marital status, education level, economic status, whether living with family, SRH, IADL, whether having chronic diseases, and district of residence. The results showed that subjects who had a higher IADL score (OR: 0.92, 95% CI: 0.85–0.99) were more likely to have a lower loneliness score. Those subjects who had a less desirable economic status, namely, those who indicated that their resources were “Just enough for daily expenses” (OR: 3.34, 95% CI: 1.40–7.96) or “Insufficient for daily expenses” (OR: 11.46, 95% CI: 3.27–40.16), felt lonelier than those who claimed to have “more than enough”. Subjects who lived alone felt lonelier than those who lived with their family (OR: 4.46, 95% CI: 1.67–12.90). With regard to districts of residence, using Shatin district as the reference, subjects living in Central and Island West, Island East, Wong Tai Sin, Kwun Tong, and Kwai Tsing were more likely to have higher loneliness scores (OR > 1). Please refer to Table 8 for details.

## 4. Discussion

Our study examined the relationship between loneliness, SRH, and IADL among older people in Hong Kong. It is the first of its kind to draw particular attention to the association between loneliness, demographic characteristics, and the district of residence of subjects within the same city. In addition, the predictors of loneliness for the older population were examined.

Most of the participants in this study reported having average to poor SRH, a medium to high level of independent living skills, and a higher level of loneliness. It is a common phenomenon for older people to have a poor perception of their health. This can be explained by the normal functional decline due to ageing as well as to the high morbidity rates from chronic illnesses. The findings are consistent with a study that reported that good SRH is related to the absence of chronic diseases [34].

Our results revealed a low negative correlation between the instrumental activities of daily living and loneliness, which is partially corroborated by previous studies. In previous studies, correlations were found between loneliness, SRH, and IADL, while better SRH among older people was associated with better IADL. The level of loneliness was significantly higher for older people who were experiencing difficulties in their self-maintenance and functional capacity, as they needed to depend on others to help them in their activities of daily living. Furthermore, their loneliness could be related to a decrease in social contact due to a decline in their physical functioning, related to impairments in physical mobility or visual acuity [9,35]. Studies have also shown that IADL reflects an individual’s physical and mental functional capacity in daily living, so that there is a significant association between poorer SRH and a decline in IADL [36,37]. For older people, good SRH was associated with social participation and the ability to go alone to distant places, which are essential components of independent living [34]. Being independent is a major factor affecting the health and quality of life of older people living in the community.

A recent longitudinal study reported that loneliness is a significant contributor to poor SRH among ageing people. Good SRH is an indicator of a decrease in loneliness or an absence of loneliness [20]. A Korean study showed that social support had a significant positive effect on perceived health status, while loneliness had a significant negative effect on perceived health status and sleep quality among community-dwelling older adults [38]. Our study partially supported Nummela’s report, as we found a low negative correlation between SRH and loneliness levels. The difference between the two studies may have been due at least in part to the different sample sizes, as 143 participants were recruited to take part in our study, far fewer than the 4272 participants in Nummela’s study. A smaller effect size would require a large sample size to detect a difference with a specified power. Despite the result, it is suggested that preventing loneliness is essential for promoting health among older people. Using a single-item scale assessing subjective self-rated health is considered essential for health care practitioners to assess the individual health status and survival rate of older people. While people having chronic diseases is regarded as a sign of poor health and is associated with loneliness [39,40]. Older people who reported greater feelings of loneliness were eight times more likely to suffer from poorer mental health than those who experienced less loneliness. A person’s health status and loneliness are interrelated, as loneliness can lead to pain, depression, and mental health problems [41].

An essential question that we examined in the current study is the association between loneliness and demographic factors. Our findings revealed that other than IADL, economic status, living status, and district of residence are predictors of loneliness, as shown in the regression analysis. Those who have a low economic status, live alone, and who live in certain districts (i.e., Central and Island West, Kwun Tong, and Kwai Tsing) experience a higher level of loneliness. Consistent with previous studies, those participants whose resources were insufficient to meet their daily expenses perceived more loneliness. Previous studies have also shown a significant association between the level of loneliness and an individual’s income, social support, and family functioning, which suggests that a minimum income should be secured for older people in the community [13,38,42]. In the current study, the result illustrated that people with the highest income reported the lowest level of loneliness and vice versa. It is easy to understand that those with fewer resources faced consumption constraints or were even unable to meet their daily living expenses. Furthermore, those who were unable to control their own money usually appeared to have limited resources, resulting in an increased level of loneliness. Moreover, economic status affects social contact, such as access to social resources, activities, and transportation, and the ability to dine with friends and make use of paid services [43,44]. It is concluded that financial strain, financial dissatisfaction, and an inability to control one’s personal finances could increase the level of loneliness of individuals.

In terms of living arrangements, married older people who were not widowed often reported a lower level of loneliness, while older people who were single or divorced reported a significantly higher level of loneliness. This implies that the absence of an intimate relationship results in a higher level of loneliness [45]. A European study found that older people in Spain and Sweden who live with their spouse/partner and who are in good health are less likely to express loneliness [46]. On the other hand, the living arrangement is not a key determinant of loneliness, as older adults who live alone are not necessarily lonely. Some older people who live with their family still feel lonely because of the inappropriate quality of the support that they receive [47]. For older people who have experienced a loss of relationships and a disruption of interpersonal or social relationships, informal social support, such as group integrated intervention programs could not only improve their social interactions but strengthen their cognitive and physical capabilities [48]. Active engagement in social and cognitive activities was found to be negatively related to both loneliness and hopelessness for Chinese older people living in the United States [49].

With regard to districts of residence, we established that the district in which an individual lives could affect their level of loneliness. Older people who lived in Shatin had a significantly lower level of loneliness than those living in Central and Island West. In Hong Kong, Shatin is more of a residential area while Central and Island West is a highly commercial district. This is inconsistent with previous findings suggesting that older people who live in rural areas have a higher likelihood of experiencing loneliness than those who live in urban areas [50]. A recent study in China argued that, with changes in social structure, urbanization is significantly associated with loneliness [51]. The differences in arguments and findings could be related to context, given that living environments differ in different regions of mainland China and Hong Kong. Nevertheless, a study revealed no association between the area of residence, whether urban or rural, and loneliness [52]. In our study, the results showed that the loneliness level of older people living in Central and Island West was significantly higher than the levels in the other five districts. The underlying reason for this could be that Central and Island West is a core urban area that is the commercial, financial, legal, and political center of Hong Kong. The Asian headquarters of many multinational companies are located there [53]. The commercial district also lacks sports stadiums, themed gardens, water sports centers, and playgrounds. Recently, a number of urban redevelopment projects have been completed to improve the living environment of the local community. Our findings are consistent with a study that older people who live in old urban districts will experience higher levels of loneliness because there are fewer facilities for leisure in such districts, requiring long travel times to access such facilities [54]. As a result, they would rather stay home than go out to engage in social activities. On the other hand, Shatin is a newly developed town with various facilities, including playgrounds, cycle tracks, a hospital, shopping centers, and cultural and recreational facilitates, along with convenient public transportation links [55]. It has a good mix of city hustle and tranquil areas, which is ideal for ageing people who prefer social connections. Despite a clear link between social connections and well-being, more research is needed to understand causal mechanisms, effect sizes, and changes over time for public health solutions.

Our results showed that the majority of the old-aged people in our study sample are lonely. Collectivist culture emphasizes the goals of the group as a whole over the needs of each individual. East Asian countries are examples of collectivist cultures [56]. Despite there being differences between social norms across Western and Eastern countries, collectivistic orientations tend to form and influence people’s emotions. A recent study conducted in five European countries reported that higher collectivism was related to lower loneliness [57]. In contrast to collectivistic values, the absence or reduced interactions in individualistic societies can result in more loneliness [16]. In applying these findings, whether it is an individualistic or collectivistic society, the best solution to reduce loneliness is through people having strong face-to-face social ties and living in a supportive society [58]. Whether the use of cell phones and social media can affect loneliness in the elderly, is an interesting topic to be considered in future studies.

### Limitations of the Study

There were some limitations to this study. Although the sample size in this study is considered adequate, the results may not be generalizable since a nonprobability sample was used after the districts were selected. Besides, the small sample size could affect the study results. As for the questionnaire, it provides a general picture of how older people perceived their SRH, their sense of loneliness, and their level of IADL. Nevertheless, it cannot provide a sufficient explanation to tap into the various associated factors, as the study was cross-sectional in design. Further well-designed studies are needed to establish cause and effect. During the data collection period, the city was affected by COVID-19; therefore, due to restrictions on outdoor activities and social engagements, the data on SRH and loneliness could have been affected.

## 5. Conclusions

This study is the first of its kind to investigate the correlation between levels of loneliness, SRH, IADL, and districts of residence among community-dwelling older people in Hong Kong. The results indicated that most older people in Hong Kong feel a relatively high level of loneliness. Older people with a low level of financial satisfaction, who are living alone, living in the central business district, and who have poor IADL are at a higher risk of loneliness. Moreover, there was a significant correlation between older people who reported having good SRH and those with a lower level of dependency in IADL. Older people who were living in a mixed commercial and residential area felt less lonely than those living in a commercial district. Future interventions should be focused on improving the social connections and facilitating the participation of older people in social activities to ease their loneliness, and thus promote their psychological well-being and ultimately improve their quality of life.

## Figures and Tables

**Table 1:** Demographic characteristics of the subjects (*n* = 143).

Characteristics	Categories	N (%)
Gender	Male	65 (45.5)
	Female	78 (54.5)
Age	65–70	49 (34.3)
	71–75	47 (32.9)
	76–80	19 (13.3)
	>80	28 (19.6)
Marital status	Married	74 (51.7)
	Single	12 (8.4)
	Divorced	17 (11.9)
	Widowed	40 (28.0)
Education level	Primary or below	66 (46.2)
	Middle School	36 (25.2)
	High School	32 (22.4)
	Undergraduate or above	9 (6.3)
Economic level	Extremely insufficient	5 (3.5)
	Insufficient for daily expenses	23 (16.1)
	Just enough for daily expenses	79 (55.2)
	More than enough	36 (25.2)
Living with	Alone	44 (30.8)
	Spouse	37 (25.9)
	Family	62 (43.4)
District of residence	Central and Western District	20 (14.0)
	Island East	22 (15.4)
	Wong Tai Sin	32 (22.4)
	Kwun Tong	21 (14.7)
	Kwai Tsing	22 (15.4)
	Shatin	26 (18.2)
Any chronic disease(s)	Yes	75 (52.4)
	No	68 (47.6)

**Table 2:** Ratings of the subjects (*n* = 143).

Variables	Categories	N (%), Median (IQR) ^1^, Mean (SD) ^2^
Self-rated Health	Very poor	7 (4.9)
	Poor	28 (19.6)
	Average	86 (60.1)
	Good	17 (11.9)
	Very good	5 (3.5)
Loneliness level		5.0 (3.0–6.0) ^1^
Instrumental Activities of Daily Living (total score)		20.77 (5.77) ^2^

Note: ^1^ IQR; interquartile range. ^2^ SD; Standard Deviation.

**Table 3:** Level of loneliness.

Score	Number of Subjects (%)
0	6 (4.2)
1	6 (4.2)
2	12 (8.4)
3	16 (11.2)
4	20 (14.0)
5	46 (32.2)
6	37 (25.9)

**Table 4:** IADL scores.

Score	Number of Subjects (%)
1–4	5 (3.5)
9	4 (2.8)
11–17	22 (15.4)
18	7 (4.9)
19–26	84 (58.7)
27	22 (14.7)

**Table 5:** The Spearman correlation coefficients (*r*) among SRH, loneliness level, and IADL (total score).

Variables	SRH	Loneliness Level	IADL (Total Score)
SRH	1	−0.292	0.375
Loneliness level		1	−0.276

**Table 6:** Differences in loneliness level (total score) among districts of residence (*n* = 143).

Characteristics	Categories	Median (IQR) ^1^	χ^2^	*p*
Living district	Central and Island West	6.00 (1.00)	18.87	0.002 *
	Island East	4.00 (2.00)		
	Wong Tai Sin	5.00 (3.00)		
	Kwun Tong	5.00 (0.00)		
	Kwai Tsing	5.00 (3.25)		
	Shatin	3.50 (4.00)		

* *p* < 0.01, ^1^ IQR; interquartile range.

**Table 7:** Test with Bonferroni corrections for pairwise comparisons.

	*p*-Value
Central and Island West vs. Island East	0.005
Central and Island West vs. Wong Tai Sin	0.02
Central and Island West vs. Kwun Tong	0.013
Central and Island West vs. Kwai Tsing	0.09
Central and Island West vs. Shatin	<0.001 *
East vs. Wong Tai Sin	0.62
East vs. Kwun Tong	0.075
East vs. Kwai Tsing	0.421
East vs. Shatin	0.116
Wong Tai Sin vs. Kwun Tong	0.533
Wong Tai Sin vs. Kwai Tsing	0.8
Wong Tai Sin vs. Shatin	0.033
Kwun Tong vs. Kwai Tsing	0.968
Kwun Tong vs. Shatin	0.003 *
Kwai Tsing vs. Shatin	0.047

* *p* < 0.01.

**Table 8:** Ordinal Regression Analysis on the predictors of loneliness.

Variables	Odds Ratio (95% CI)	*p*-Value
SRH ^1^	0.64 (0.39–1.05)	0.078
IADL ^2^ (total score)	0.92 (0.85–0.99)	0.034 *
Gender		
Male	1.37 (0.68–2.75)	0.373
Female	1.00 ^Ref^	
Age group		
65–70	0.80 (0.24–2.66)	0.722
71–75	0.40 (0.13–1.23)	0.110
76–80	0.52 (0.15–1.81)	0.300
>80	1.00 ^Ref^	
Marital status		
Married	2.22 (0.71–6.96)	0.170
Single	0.53 (0.11–2.61)	0.431
Divorced	1.80 (0.53–6.16)	0.349
Widowed	1.00 ^Ref^	
Education level		
Primary or below	0.75 (0.14–3.91)	0.731
Middle school	2.15 (0.42–11.01)	0.356
High school	4.70 (0.86–25.59)	0.074
Undergraduate or above	1.00 ^Ref^	
Economic status		
Extremely insufficient	1.92 (0.28–13.16)	0.508
Insufficient for daily expenses	11.46 (3.27–40.16)	* <0.001
Just enough for daily expenses	3.34 (1.40–7.96)	* 0.007
More than enough	1.00 ^Ref^	
Living with		
Alone	4.64 (1.67–12.90)	* 0.003
Spouse	0.63 (0.23–1.67)	0.349
Family	1.00 ^Ref^	
Any chronic disease		
Presence of chronic disease	1.73 (0.77–3.91)	0.186
None	1.00 ^Ref^	
District of Residence		
Central and Island West	21.33 (4.81–94.51)	* <0.001
Island East	3.34 (1.02–10.99)	* 0.047
Wong Tai Sin	5.00 (1.63–15.36)	* 0.005
Kwun Tong	5.66 (1.51–21.16)	* 0.010
Kwai Tsing	6.33 (1.56–25.77)	* 0.010
Shatin	1.00 ^Ref^	

^1^ SRH; Self-rated health. ^2^ IADL; Instrumental Activities of Daily Living. Ref = reference group. * *p* < 0.05

## Data Availability

The data presented in this study are available on reasonable request from the corresponding author.
